# 3D Printed Polymer Piezoelectric Materials: Transforming Healthcare through Biomedical Applications

**DOI:** 10.3390/polym15234470

**Published:** 2023-11-21

**Authors:** Fawad Ali, Muammer Koc

**Affiliations:** Division of Sustainable Development, College of Science and Engineering, Hamad Bin Khalifa University, Qatar Foundation, Doha 34110, Qatar; mkoc@hbku.edu.qa

**Keywords:** polymer piezoelectric materials, 3D printing, biomedical applications, tissue engineering

## Abstract

Three-dimensional (3D) printing is a promising manufacturing platform in biomedical engineering. It offers significant advantages in fabricating complex and customized biomedical products with accuracy, efficiency, cost-effectiveness, and reproducibility. The rapidly growing field of three-dimensional printing (3DP), which emphasizes customization as its key advantage, is actively searching for functional materials. Among these materials, piezoelectric materials are highly desired due to their linear electromechanical and thermoelectric properties. Polymer piezoelectrics and their composites are in high demand as biomaterials due to their controllable and reproducible piezoelectric properties. Three-dimensional printable piezoelectric materials have opened new possibilities for integration into biomedical fields such as sensors for healthcare monitoring, controlled drug delivery systems, tissue engineering, microfluidic, and artificial muscle actuators. Overall, this review paper provides insights into the fundamentals of polymer piezoelectric materials, the application of polymer piezoelectric materials in biomedical fields, and highlights the challenges and opportunities in realizing their full potential for functional applications. By addressing these challenges, integrating 3DP and piezoelectric materials can lead to the development of advanced sensors and devices with enhanced performance and customization capabilities for biomedical applications.

## 1. Introduction

For many years, researchers in the field of biomaterials have been primarily focused on developing biocompatible materials that can interact effectively with the biological system. These materials, known as bioactive materials, have been a significant area of interest. However, in recent times, there has been a growing fascination with a new type of material called bio-smart material. These materials possess the ability to respond to external triggers and imitate the behavior of natural biological tissues. They are also referred to as reactive, responsive, or intelligent functional materials. The unique characteristic of bio-smart materials is their active participation, where they can sense, diagnose, and react to external stimuli. Such stimuli may include changes in temperature, pH, specific chemicals, or electric and magnetic fields. When exposed to these stimuli, the smart material undergoes controlled changes in its properties. The potential applications of smart materials in the development of advanced biomedical devices, temporary implants, and drug delivery systems have generated immense interest among scientists and clinicians. A range of such types of materials are currently available, but the most promising in the field of biomedicine are piezoelectric materials [1,2,3,4,5,6].

Piezoelectric materials have gained significant attention in biomedical applications due to their unique ability to convert mechanical energy into electrical energy and vice versa. The phenomenon of piezoelectricity was first discovered by Pierre and Jacques Curie in the late 19th century [7], when they observed that certain crystals, such as quartz, generate an electric charge when subjected to mechanical stress. Since then, piezoelectric materials have found diverse applications in various fields, including biomedical engineering [3,8,9,10,11,12,13]. In the context of biomedical applications, piezoelectric materials offer several advantages. They exhibit excellent sensitivity, allowing for the precise detection and measurement of mechanical forces, vibrations, and pressure. This property makes them highly suitable for sensing applications in biomechanics, such as monitoring physiological parameters like heart rate, respiratory rate, and blood pressure [14,15,16]. A recent study demonstrates a process for creating complex, manganese-doped zinc sulphide/zinc calcium oxysulfide-based chemically stable ceramic parts with low surface roughness, thus expanding their potential applications in 3D printing technology for damage detection and healthcare applications [17,18].

Piezoelectric materials also play a crucial role in medical imaging and diagnostics [19,20,21]. For instance, in ultrasound imaging, piezoelectric transducers generate and receive ultrasonic waves [22], enabling non-invasive imaging of internal organs and tissues. The ability of piezoelectric materials to convert electrical signals into mechanical vibrations and vice versa makes them essential components in ultrasound probes. Moreover, piezoelectric materials have been employed in therapeutic applications. They are used in devices for targeted drug delivery and tissue stimulation [23]. By applying electrical voltage to the piezoelectric material, it undergoes mechanical deformation, facilitating the controlled release of drugs or promoting tissue regeneration through mechanical stimulation.

Another exciting area of research is the development of piezoelectric energy harvesters [24]. These devices utilize biological movements as a source of mechanical energy, such as walking or the beating of the heart, to generate electrical power. This technology holds immense potential for self-powered implantable biomedical devices, eliminating the need for external power sources or battery replacements.

While traditional piezoelectric materials, such as quartz and ceramics [25,26], have been widely used in biomedical applications, the emergence of polymer-based piezoelectric materials has opened up new possibilities [27,28]. Polymer piezoelectric materials offer advantages such as flexibility, lightweightness, biocompatibility, and ease of processing [29]. They can be fabricated into various shapes and forms, enabling their integration into flexible and wearable devices.

In recent years, extensive research has focused on exploring the potential of polymer piezoelectric materials in areas such as biomedical sensing, energy harvesting, imaging, drug delivery, and tissue engineering. Polymeric materials and their composites have largely been used for biomedical applications [12]. The development of novel polymer piezoelectric materials with enhanced performance and tailored properties continues to drive advancements in the field of biomedical engineering, opening up exciting avenues for the development of innovative and patient-centric healthcare technologies.

The 3D printing of poly piezoelectric materials, also known as 3D printing of piezopolymers, is an emerging field with great potential in biomedical applications. Multiple 3D printing techniques have been employed to create piezoelectric materials, including FDM (material extrusion), stereolithography (powder bed fusion), and selective laser sintering (SLS). Using the processes, piezoelectric structures can be produced after high-temperature sintering to achieve piezoelectric characteristics [29,30,31,32].

## 2. Piezoelectric Phenomena

**Figure 1:**
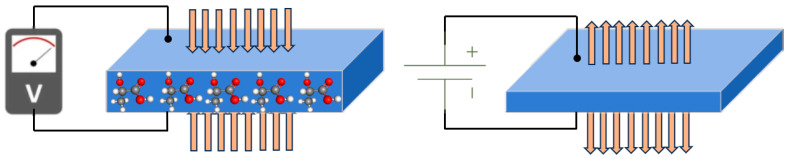
Graphical representation of direct and converse piezoelectric effect.

Piezoelectric phenomena refer to the unique properties exhibited by certain materials that can generate an electric charge in response to mechanical stress or deformation, and vice versa. These materials are called piezoelectric materials, and they possess a special crystal structure or molecular arrangement that allows for the generation and conversion of electrical and mechanical energy. When a piezoelectric material is subjected to mechanical stress or strain, such as compression, tension, or bending, the material undergoes a distortion at the atomic or molecular level. This distortion causes a separation of positive and negative charges within the material, resulting in the generation of an electric potential across the material. This phenomenon is known as the direct piezoelectric effect. Conversely, when an electric field is applied to a piezoelectric material, it experiences a change in shape or deformation. This is referred to as the converse piezoelectric effect. The electric field causes the positively and negatively charged particles within the material to shift, resulting in a mechanical strain or displacement. The direct and converse piezoelectric effect is depicted in Figure 1 and described using the following constitutive equations:

(1) D=dT+ε E (Direct effect) 

(2) $$X=sT+dE\;\left(Converse\;effect\right)$$

where *D* is the relative electric displacement, *d* is the piezoelectric coefficient, *T* is the stress, *ε* is the dielectric constant of the material, *E* is the electric field, *X* is the strain, and *s* is the mechanical compliance.

## 3. Fundamentals of Polymer Piezoelectric Materials

Polymer piezoelectric materials are organic polymers that possess the ability to generate an electric charge in response to mechanical stress or strain and, conversely, to deform when subjected to an electric field. This phenomenon is based on the piezoelectric effect, which arises from the asymmetrical distribution of electric charges within the polymer structure [33,34].

Polymer Selection: The choice of polymer is crucial and depends on factors such as desired piezoelectric performance, biocompatibility, mechanical properties, processing methods, and stability. Commonly used polymer materials include polyvinylidene fluoride (PVDF), poly(vinylidene fluoride-co-trifluoroethylene) (PVDF-TrFE), poly(lactic acid) (PLA), and poly(vinyl alcohol) (PVA), among others.

Piezoelectric Properties: Key properties of polymer piezoelectric materials include the piezoelectric coefficient (*d*), which quantifies the magnitude of electric charge generated under stress; the dielectric constant (ε), which influences the energy storage capacity; and mechanical properties such as elasticity, flexibility, and resilience.

Electrical Poling: To enhance the piezoelectric response of polymer materials, an electrical poling process is often employed. It involves subjecting the material to a high electric field while above its glass transition temperature, aligning the polymer chains, and creating a permanent piezoelectric effect.

Biocompatibility: Biomedical applications require polymer piezoelectric materials to be biocompatible, meaning they should be non-toxic, non-immunogenic, and exhibit minimal adverse effects on living tissues or organisms. Evaluating and ensuring the biocompatibility of these materials is crucial for their safe integration into healthcare devices.

Stability and Longevity: Polymer piezoelectric materials should exhibit long-term stability, maintaining their piezoelectric properties over extended periods of use. The durability of these materials is essential for their reliable performance in biomedical applications.

Understanding these fundamentals is essential for the development, design, and optimization of polymer piezoelectric materials specifically tailored for biomedical applications, enabling advancements in sensing, energy harvesting, imaging, drug delivery, and other healthcare technologies.

## 4. Polymer Piezoelectric Materials

The use of conventional piezoceramics in applications that demand flexible devices has been restricted due to their brittleness and rigidity. As a result, there has been a growing interest in flexible piezoelectric polymers that exhibit excellent piezoelectric properties. This interest has been particularly prominent in the biomedical field. Polymer piezoelectric materials are a class of materials that exhibit piezoelectric properties while being composed primarily of organic polymers. Unlike traditional piezoelectric materials, polymer piezoelectric materials offer unique advantages, including flexibility, lightweight nature, biocompatibility, and ease of processing. These properties make them attractive for a wide range of applications, particularly in biomedical fields.

**Figure 2:**
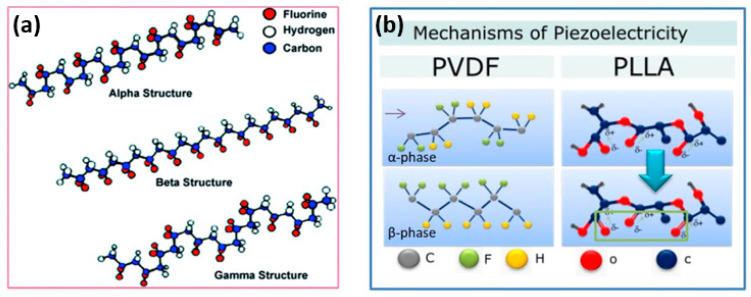
(**a**) Chain conformation for α, β, and γ crystalline phases of PVDF [40] and (**b**) Piezoelectric mechanism in polymers [28].

Polymer piezoelectric materials can be engineered by incorporating piezoelectric polymers or by poling non-piezoelectric polymers to induce piezoelectric behavior. Some commonly used piezoelectric polymers include polyvinylidene fluoride (PVDF), poly(vinylidene fluoride-co-trifluoroethylene) (PVDF-TrFE), poly(lactic acid) (PLA), poly(vinyl alcohol) (PVA), and their copolymers. These polymers exhibit inherent piezoelectricity due to their molecular structure, which allows for the generation and conversion of electrical signals in response to mechanical stress. Polyvinylidene fluoride (PVDF) and its copolymers have garnered significant attention in research due to their exceptional piezoelectric properties, excellent chemical resistance, thermal stability, favorable processability, and mechanical characteristics when compared to other piezoelectric materials. PVDF can exist in five different piezoelectric crystal phases, with the β-phase exhibiting the highest piezoelectricity [35,36,37]. The arrangement of PVDF molecular dipoles, represented by CH_2_-CH_2_ groups, can adopt various chain conformations, as shown in Figure 2a. The presence of strongly electronegative fluorine atoms contributes to the formation of a non-zero dipole moment within the crystal structure [38,39]. This molecular arrangement is observed in the β, γ, and δ phases, with the β-phase having the strongest dipole moment due to the all-trans conformation [40]. The α phase is typically obtained directly from the melt through crystallization. The β phase, characterized by an all-trans planar zigzag conformation, results in a substantial dipole moment. Additionally, the presence of additive dipole moments contributes to the generation of a large spontaneous polarization. In contrast, the piezoelectric effect of the γ phase in PVDF is weaker compared to the β phase, primarily due to the presence of a gauche bond occurring in every fourth repeating unit [41]. In the case of alternative chain conformations (TGTG-, T3GT3G-), parallel dipole arrangements can be seen in the δ and γ phases, respectively, resulting in lower polarity. When the same conformations exhibit antiparallel chain dipole arrangement, it leads to a net dipole moment of zero, as in the α phase [42,43].

In contrast to inorganic piezoceramics like PZT, poly-L-lactic acid (PLLA) possesses lower piezoelectricity; however, as a film, it exhibits a significant piezoelectric shear constant. Figure 2b (right) illustrates the thermodynamic equilibrium of a polylactic acid (PLLA) chain in its α-crystalline form. CO dipoles are statistically distributed along the main chain. When the α-crystalline form is subjected to stretching, it undergoes a transformation to the piezoelectric β-crystal modification, aligning the dipoles along the stretching direction. The electrospinning process of fiber formation can also induce alignment of the CO bonds, resulting in piezoelectric behavior in PLLA. Due to its flexibility, PLLA is suitable for applications on mobile devices [44]. Additionally, as a biodegradable and biocompatible polymer, PLLA holds great potential for future applications such as biosensors and actuators [45].

Most piezoelectric biomaterials belong to the category of natural polymers derived from polysaccharides and polypeptides, including cellulose, chitin, chitosan, amino acids, collagen, and peptides [46]. The underlying principles of piezoelectricity in biomaterials stem from their intricate dipolar properties and dipole-dipole interactions facilitated by complex hydrogen bonding networks, which exhibit varying levels of self-assembly and hierarchical organization. These biomaterials possess a crystalline structure that forms a network of unit cell dipoles, similar to the mechanisms observed in conventional inorganic piezoelectric materials such as piezoceramics. This crystalline structure, characterized by orthorhombic and monoclinic space groups, is responsible for the piezoelectric properties exhibited by biomaterials [46]. Chitosan, a biodegradable natural polymer, is also recognized as a piezoelectric material. It is derived from chitin through a deacetylation process. Chitosan is a linear polysaccharide composed of the rancdom association of two distinct β(1-4) linked structural units: -amino-2-deoxy-D-glucose and N-acetyl-2-amino-2-deoxy-D-glucose [47]. To ensure successful conversion from chitin to chitosan, the degree of deacetylation should reach approximately 50% [48]. Chitosan possesses favorable properties such as biocompatibility, biodegradability, and non-toxicity and can be easily formed into films, rendering it suitable for various applications in the pharmaceutical, cosmetic, and immobilization fields [49].

**Figure 3:**
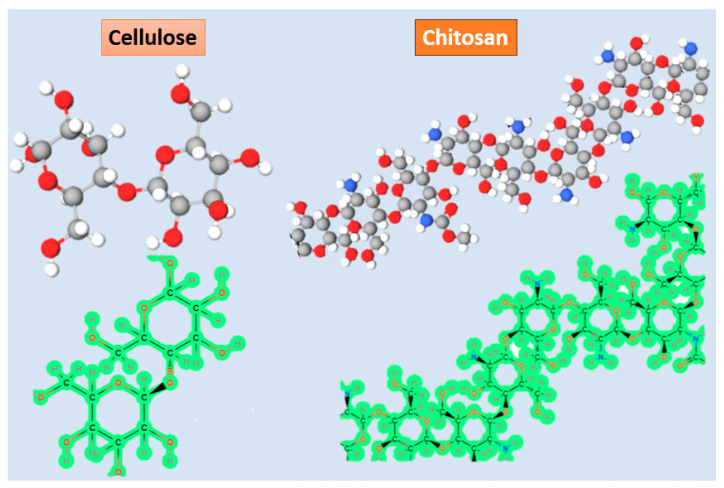
Schematic view of structural formula and 3D representation of cellulose and collagen.

Biopolymer-based piezoelectric materials consisting of cellulose and chitin are depicted in Figure 3. Cellulose, which constitutes a significant portion of biopolymers on Earth, exhibits piezoelectric properties [50]. Its remarkable biocompatibility, tensile strength, and ability to mimic the biological environment make cellulose highly appealing for biomedical applications. However, the small pore size and densely packed fiber structure of cellulose restrict cell penetration. This limitation is addressed by the use of progens, which facilitate the creation of suitable pores. Additionally, cellulose plays a crucial role in enhancing cellular adhesion, including chondrocytes, osteocytes, endothelial cells, and smooth muscle cells [51]. Considering all these mentioned characteristics, it is reasonable to assert that cellulose serves as a suitable piezoelectric material for tissue engineering applications in both bone and cartilage [52,53]. Moreover, in a particular study, a cellulose-based scaffold was employed for skin tissue engineering and wound healing [54].

Collagen, a piezoelectric biomaterial, exhibits a piezoelectric coefficient ranging from 0.2 to 2.0 pC/N [55]. Its favorable characteristics, including suitable cell adhesion, excellent biocompatibility, hydrophilicity, low antigenicity, and absorbability in the host body, make collagen an attractive biomaterial for tissue engineering applications [56]. As a crucial biological protein, collagen plays a vital role in the extracellular matrix (ECM) of various tissues, such as cartilage, tendon, bone, blood vessels, and teeth, providing mechanical support [57]. Numerous studies have utilized collagen-based scaffolds. For instance, ZhiCai et al. [56] developed a collagen-based scaffold for bone tissue engineering and found that although the fabricated scaffold showed poor mechanical properties in vivo, electrospun nanofibrous scaffolds enhanced the mechanical properties and could serve as favorable implants for bone healing. Additionally, in another study, PLGA-collagen hybrid scaffolds with controlled pore structures were employed for cartilage tissue engineering [58]. Furthermore, the efficiency of collagen-calcium phosphate composite scaffolds was demonstrated [57], revealing 96% cartilage formation 20 weeks after implantation of the scaffold. However, collagen usage in tissue engineering is restricted by challenges such as poor mechanical stiffness, rapid degradation, and toxicity associated with the addition of crosslinking agents. Crosslinking agents are incorporated into collagen to reduce the biodegradation rate. Physical and chemical approaches exist for crosslinking, including dehydrothermal treatment (DHT), photooxidation, and UV irradiation as physical methods that enhance the bio-durability of collagen-based scaffolds without cytotoxicity [57]. However, chemical methods remain crucial for achieving the necessary level of crosslinking in most cases [59]. Common chemical crosslinking treatments involve the incorporation of synthetic peptides mimicking collagen triple helices [60], as well as the use of formaldehyde, glutaraldehyde, and other agents. Glutaraldehyde is the most traditional and conventional crosslinking agent employed for collagen-based scaffolds [61]. However, the cytotoxicity associated with these chemical crosslinking agents poses a challenge. Consequently, researchers have sought the utilization of bioactive molecules that can stabilize collagen without causing toxicity. In one study, Terminalia chebula fruits drew attention due to their rich of polyphenol extracts. Polyphenols were used as a crosslinking agent, and polyphenol-treated collagen sheets (PPCS) were fabricated to simultaneously enhance the physical and chemical properties of the scaffold for wound healing applications, minimizing after-effects such as cytotoxicity and rapid biodegradation [59].

Chitin, depending on its source, exhibits piezoelectric coefficients ranging from 0.2 to 1.5 pC/N [55]. Chitosan, a biopolymer derived from the deacetylation of chitin, is considered a cost-effective and environmentally-friendly piezoelectric material [48]. However, its relatively low piezoelectricity has limited its use as a primary material for fabricating scaffolds in tissue engineering applications. Instead, chitosan is predominantly utilized in tissue engineering for its favorable biocompatibility and biodegradability rather than its piezoelectric properties. Often, it is blended with other biomaterials [23,62]. In certain studies [48,62], chitosan has been employed in the fabrication of biodegradable sensors. Its incorporation has demonstrated an enhancement in the piezoelectricity of the sensors, which is a crucial factor in their sensor functionality.

In biomedical applications, polymer piezoelectric materials have shown great promise. They have been used in sensors for detecting and measuring physiological parameters such as pressure, strain, and motion. Polymer piezoelectric materials have also been incorporated into wearable devices for monitoring health and wellness, as well as in implantable devices for energy harvesting, enabling self-powered biomedical systems. Additionally, they have been utilized in medical imaging, drug delivery systems, tissue engineering, and even as actuators for controlled mechanical stimulation.

The development of novel polymer piezoelectric materials continues to drive advancements in the field. Researchers are exploring new materials, fabrication techniques, and device designs to improve their performance, stability, and biocompatibility. Polymer piezoelectric materials hold significant potential for transforming healthcare technologies by offering innovative solutions that are flexible, lightweight, and well-suited for integration into biomedical applications.

## 5. Polymer Composite Piezoelectric Materials

To address the issue of piezopolymers exhibiting low piezoelectric coefficients, researchers have proposed the use of piezocomposites, also known as piezonanocomposites, which involve combining piezopolymers with high piezoelectric coefficient inorganic materials [63]. These piezoelectric composites demonstrate improved piezoelectric properties compared to non-composite piezopolymers [64]. By utilizing piezoelectric composites, it becomes possible to overcome the temperature limitations associated with piezopolymers, as well as the inherent brittleness of inorganic bio-piezomaterials, while enabling large-scale manufacturing capabilities In one study conducted by, a novel bio-piezoelectric composite was developed for use as bone cement by incorporating barium titanate (BaTiO_3_ [BT]) particles into the biocompatible polymer polymethyl methacrylate. The inclusion of BT particles was found to enhance the osteoinductivity of the cement. Furthermore, the addition of graphene, which increases conductivity and dielectric constant, allowed for the attainment of a piezoelectric coefficient comparable to that of human bones even with a low BT content in the cement. Such bio-piezoelectric composites were found to promote cell proliferation, with an increased cell count corresponding to higher piezoelectric coefficients. Another study involved fabricating a piezoelectric composite using the electrospinning technique and conventional piezoelectric polymers, namely polyvinylidene fluoride and silk fibroin. This approach successfully maintained the bio-piezoelectric characteristics of the composite while addressing the limitations associated with pure PVDF electrospun mats, thereby improving their physical properties. Silva et al. conducted research on collagen-hydroxyapatite composites to examine their piezoelectric and dielectric properties [65]. The results demonstrated that composites incorporating nanocrystalline hydroxyapatite exhibited superior performance (d14 = 0.040 pC/N) compared to composites with commercially available hydroxyapatite (d14 = 0.012 pC/N).

Similarly, the co-polymer of PVDF (poly(vinylidene fluoride-co-trifluoroethylene)) (P(VDF-TrFE)) is also extensively studied as a piezoelectric material for nano-generators due to its biocompatibility and ease of fabrication [24,66,67]. Gui et al. developed piezoelectric nanogenerators (PENGs) by incorporating interdigital Au electrode nanofibers (IDT) within P(VDF-TrFE) nanofibers [68]. The integration of IDTs served a dual purpose: they acted as parallel electrodes for collecting nanofibers during the electrospinning process and as electrodes to gather charges generated by the PENG. The inclusion of a well-defined cylindrical cavity structure in the PENG enhanced its output performance by inducing deformation in the P(VDF-TrFE) nanofibers when subjected to low-frequency input forces. The resulting PENG achieved an output voltage of 5 V and an output current of 875 nA [68].

**Figure 4:**
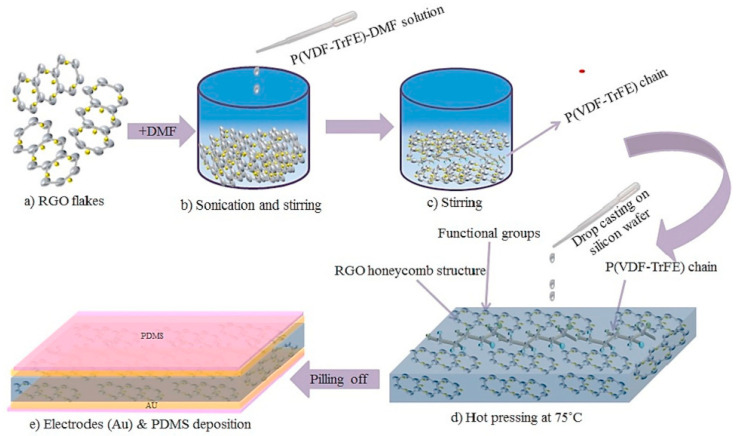
Fabrication process of P(VDF-TrFE)-RGO thin film. (**a**) RGO flakes (**b**) Sonication and Sintering (**c**) Sintering of P(VDF-TrFE) chain (**d**) hot pressing and (**e**) electrodes deposition [69].

Furthermore, the introduction of reduced graphene oxide (RGO) without the need for the poling process improved the energy harvesting capabilities of a P(VDF-TrFE) matrix. A PENG composed of 0.1 wt% P(VDF-TrFE)-RGO exhibited the highest open circuit voltage of 2.4 V and a short circuit current of 0.8 mA [69]. To create the flexible piezoelectric nanogenerator using P(VDF-TrFE)-RGO, the process began by placing the Si substrate on a hot plate set at 75 °C. Subsequently, all five samples were carefully drop-casted onto the Si substrate and subjected to a five-hour heat treatment at 75 °C. Following this heating process, the dried films were delicately peeled off from the Si substrate, and gold (Au) electrodes were applied to both sides of the thin films using a magnetron sputtering technique. Next, platinum (Pt) wires were affixed to the electrodes using silver (Ag) epoxy. Finally, for packaging purposes, a polydimethylsiloxane (PDMS) solution was drop-casted over the entire device. The complete synthesis steps and a schematic representation of the thin film are depicted in Figure 4.

Incorporating magnesium oxide nanoparticles (MgO NPs) as fillers in P(VDF-TrFE) proved effective in enhancing the piezoelectric and ferroelectric properties. Additionally, MgO NPs improved the breakdown electric field and reduced leakage current [70,71]. Recently, a MgO/P(VDF-TrFE) film was fabricated through a casting method, resulting in a PENG with exceptional mechanical properties. The incorporation of 2 wt% MgO NPs in P(VDF-TrFE) led to a significant enhancement of almost 50% in piezoelectric and polarization responses, with the PENG on flexible substrates exhibiting an open circuit voltage of 2 V, surpassing that of pure P(VDF-TrFE) films [70]. P(VDF-TrFE) nanofibrous scaffolds (NFSs) have gained considerable interest in wound healing and tissue engineering applications. Implantation of polarized PVDF-TrFE NFSs beneath the skin of a rat’s leg resulted in measurements of 6 mV for output voltage and approximately 6 µA for output current. Due to the enhanced piezoelectric effect and favorable cytocompatibility, fibroblast cell proliferation rates stimulated by the surface charges from poled electrospun P(VDF-TrFE) NFSs exhibited a significant enhancement, reaching up to 1.6 times greater than that of the unpoled counterpart [69].

## 6. Polymer Piezoelectric Materials Biomedical Applications

Polymer piezoelectric materials have gained significant attention in biomedical applications due to their advantageous properties such as flexibility, biocompatibility, and ease of processing. These materials can convert mechanical energy into electrical signals and vice versa, making them suitable for various biomedical applications. Here are some examples:

## 7. Tissue Engineering

Biocompatible, biodegradable, small, and flexible tissue stimulators have found diverse applications in the field of biomedicine. Recent studies have explored the use of organic piezoelectric actuators in tissue engineering scaffolds to facilitate tissue regeneration.

Frias et al. conducted experiments employing a PVDF actuator to validate the feasibility of using piezoelectric materials to directly strain bone cells through the converse piezoelectric effect [72,73]. The actuator design comprised a thin PVDF film with silver ink electrodes printed on both sides. To enhance osteoblast adhesion to the device surface and provide electrical insulation, the PVDF surface was coated with a poly(methyl methacrylate) (PMMA)/bone-like apatite layer. Osteoblasts were cultured on the piezoelectric material surface, and the researchers investigated the cellular response of the osteoblasts. The results indicated that both static and dynamic substrates influenced cell viability and proliferation.

Damaraju et al. presented a method for constructing flexible and three-dimensional fibrous scaffolds using piezoelectric materials, which were employed to induce the differentiation of human mesenchymal stem cells [74]. The researchers used a testing device to measure the electrical output of the scaffolds. Scaffolds with high-output voltage facilitated osteogenic differentiation, while those with low voltage output promoted chondrogenic differentiation. The findings indicated that electromechanical actuation resulted in greater cell differentiation compared to mechanical actuation alone.

Mota et al. examined the utilization of piezoelectric nanocomposites made of PVDF fibers and barium titanate nanoparticles (BTNPs) for cochlear stimulation. They employed electrospun ultrafine fibers of BTNP/PVDF as novel electromechanical transducers for restoring cochlear function [75]. The biocompatibility of the material was assessed by seeding OC-k3 epithelial cells into wells containing nanocomposite fibers. The presence of intact nuclei in the surrounding areas indicated cytocompatibility. Furthermore, the BTNP/PVDF fibers promoted the proliferation of neural cells under dynamic culture conditions. Overall, these findings suggest that nanostructured piezoelectric materials can enhance material performance by facilitating favorable interactions with tissues at the cellular level.

Wang et al. investigated the influence of electrospinning parameters on the piezoelectric properties of PVDF nanofiber actuators [76]. They fabricated PVDF nanofibers using an electrospinning setup and then applied pressure to achieve a smooth surface. Au electrodes were sputtered onto both surfaces of the pressed fibrous mat, which was subsequently poled in a silicon oil bath under an electric field to enhance its piezoelectricity. The optimized electrospinning conditions yielded an actuator that was used for implanted energy harvesting in rats. The electrospun PVDF-TrFE nanofibers exhibited excellent biocompatibility and a significant piezoelectric effect, leading to perfect development and a 1.6-fold increase in the proliferation rate of fibroblast cells along the fiber direction.

The use of wireless stimulation to induce piezoelectricity in polymers to promote the differentiation of neuronal cells offers a promising approach for contactless and controlled neuroregenerative therapies. Hoop et al. conducted an in vitro demonstration of the effect of ultrasonically stimulated piezoelectric β-PVDF on neurite generation in PC12 cells [77]. They coated a commercially purchased PVDF membrane with gold electrodes and applied ultrasonic waves to induce mechanical deformations. The results indicated that ultrasound could induce polarization in the piezoelectric polymers, initiating the differentiation of PC12 cells. This study highlights the potential of combining ultrasonic actuation and piezoelectric polymers for neuronal cell differentiation, emphasizing the need for further investigation in this area.

Recent advancements in tissue engineering have demonstrated the utilization of PVDF scaffolds to preserve the contractility of cardiomyocytes and facilitate cell-to-cell communication [78]. In this particular study, a proposed scaffold was developed by incorporating piezoelectric microfibers of PVDF-TrFE (referred to as PIEZO) onto a polycaprolactone (PCL) magnetic nanofilm (MNF). The fabrication process involved depositing electrospun nanofibers of PIEZO onto a spin-coated solution of PCL to create the MNF+PIEZO scaffold. Piezoresponse force microscopy (PFM) revealed a maximum piezoelectric constant of d14 = 11.1 pm V^−1^. The reported outcomes indicated that cardiac cells cultured on the MNF+PIEZO scaffold exhibited enhanced contractility for a minimum of 12 days. Overall, the scaffold demonstrated favorable attachment to rat and human cardiac cells. However, further investigations are warranted to explore the impact of piezoelectric materials on cardiomyocyte function.

**Figure 5:**
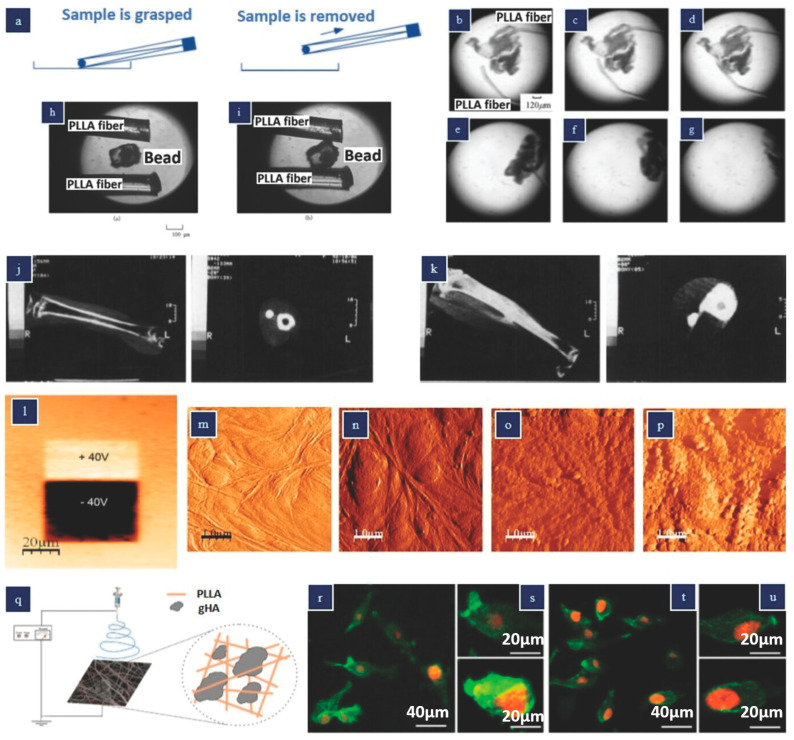
Actuators and stimulators made from poly-lactic acid (PLLA) (**a**) Twizeer (**b**) Images depicting the manipulation of a thrombosis sample utilizing PLLA fibers under voltage control, and (**e**–**g**) extraction of a thrombosis sample. (**a**–**g**) Reproduced with permission [69]. (**h**) liberating a bead and (**i**) seizing a bead utilizing PLLA fibers under applied voltage control. (**h**,**i**) Reproduced with permission [80] Copyright 2004, Taylor & Francis Group. (**j**,**k**) X-ray computed tomography images of cats’ tibiae 8 weeks post-implantation of rods composed of various materials: (**j**) untreated PLLA; (**k**) stretched PLLA (stretch ratio = 4). (**j**,**k**) Reproduced with permission [83] Images of PLLA films obtained after polarization with a DC bias of ±40 V (**m**–**p**) Surface topography visuals of PLLA thin film in AFM tapping mode in liquid: (**m**) unpoled region prior to protein adsorption, (**n**) unpoled region after protein adsorption, (**o**) positively poled region after protein adsorption, and (**p**) negatively poled region after protein adsorption. (**l**–**p**) Reproduced with permission [84] (**q**) Schematic of the electrospinning apparatus used to produce the gHA–PLLA membranes (**r**–**u**) Representative confocal laser scanning microscopy (CLSM) images of MG-63 osteoblastic cells cultured on PLLA (**r**,**s**) and gHA–PLLA membranes (**t**,**u**) for 24 h. Reproduced with permission [85].

Tajitsu et al. conducted a series of studies where they utilized the piezoelectric properties of PLLA (poly-L-lactic acid) to create a biodegradable tweezer designed for treating thrombosis [79,80,81,82]. This tweezer can be inserted into the body using a catheter. Figure 5a illustrates the operating principle of this straightforward tweezer design. By applying an external AC voltage, the PLLA fibers, produced through dry jet spinning, can be actuated. The researchers demonstrated the tweezer’s effectiveness in grasping blockages caused by thrombosis within a blood vessel, as depicted in Figure 5b–d, and subsequently removing them, as shown in Figure 5e–g. The tweezer also showed potential for releasing and grasping silica beads, as presented in Figure 5h,i. The PLLA tweezers possess notable traits such as biocompatibility, biodegradability, and high sensitivity, making them suitable for various applications in cellular biology, tissue engineering, nanomedicine, and cell delivery.

There have been several efforts to utilize the biocompatible and biodegradable piezoelectric polymer PLLA (poly-L-lactic acid) as a tissue stimulator to enhance cell proliferation and differentiation. In the case of feline tibiae, implanted-drawn (piezoelectric) PLLA rods within the intramedullary cavity resulted in significantly greater callus formation compared to undrawn (non-piezoelectric) PLLA rods [83]. This observation suggests that increasing the draw ratio of the PLLA rod improves fracture healing by promoting increased callus formation. Consequently, drawn PLLA may serve as improved fracture fixation devices since they are resorbable in the body, eliminating the need for a second surgery.

The beneficial effects of piezoelectric PLLA on tissue regeneration may be attributed to its polarized nature, which leads to enhanced protein adsorption, cellular adhesion, and proliferation [86]. Figure 5l illustrates a PFM (piezoresponse force microscopy) image of nonpoled, positively poled, and negatively poled PLLA films, while Figure 5m–p presents atomic force microscopy (AFM) results for different PLLA films. It is evident that the poled PLLA samples exhibit significantly higher protein adsorption on their surfaces. Notably, negatively charged PLLA demonstrates increased adhesion and proliferation of cells. The protein adsorption test reveals that surface charges can influence the conformation or orientation of adsorbed proteins, potentially exposing or obstructing their cell-binding domains [34].

Santos et al. carried out a study where they produced hybrid microcomposite electrospun membranes using PLLA as the matrix and glass-reinforced hydroxyapatite (gHA) micrometer particles as a filler material, aiming to enhance bone regeneration compared to PLLA membranes alone [85]. It is worth mentioning that electrospinning PLLA nanofibers has been shown to yield piezoelectric properties. The electrospinning setup employed to obtain the PLLA and gHA–PLLA membranes is presented in Figure 5q. Osteoblastic cells were seeded on the composite membranes, and at day 1, enhanced cell spreading was observed on the gHA–PLLA samples compared to the PLLA samples, as illustrated in Figure 5r–u. The bone-bonding ability analysis revealed that both sample types induced HA (hydroxyapatite) crystal nucleation, but the gHA microcomposite exhibited better organization of the F-actin cytoskeleton and increased alkaline phosphatase activity. These findings suggest that the gHA–PLLA membranes hold promise for bone healing applications.

### Skin Healing

When the skin is injured, an electrical field is generated as a result of the transepithelial potential (TEP) differences at the damaged epithelial layer, caused by varying ionic gradients. This electrical field plays a crucial role in promoting wound healing and skin regeneration by enhancing cell regeneration and facilitating processes such as fibroblast proliferation, myofibroblast differentiation, keratinocyte migration, and angiogenesis [87]. Cells are known to be electrically sensitive, and external electrical fields can influence their directional migration towards the anode or cathode [88]. However, previous methods of supplying electricity required large devices and hospitalization, posing challenges. By utilizing piezoelectric materials instead, the process of skin regeneration becomes faster, more cost-effective, and less time-consuming [89].

**Figure 6:**
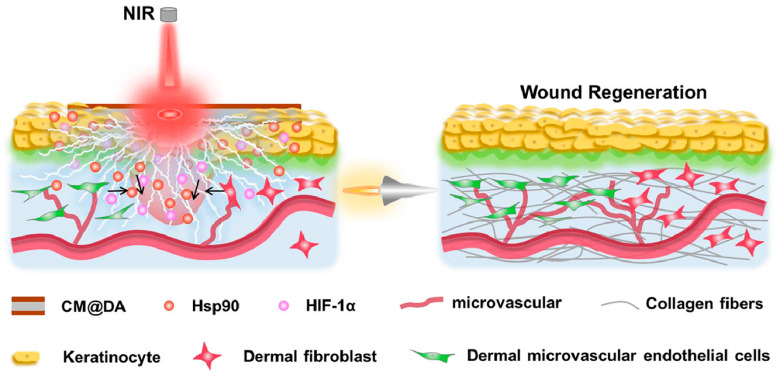
The use of piezoelectric materials for wound healing [92].

The in vivo experiment using a rat wound model confirms that CM@DA combined with NIR effectively accelerates wound regeneration by generating electric stimulation and heat. Further investigation reveals that CM@DA enhances wound healing through the involvement of heat shock protein 90 (Hsp90) and hypoxia-inducible factor 1α (HIF-1α), suggesting its potential as a future wound dressing in clinical applications, as shown in Figure 6. A study focused on investigating a piezoelectric dermal patch and found that it significantly improved the skin regeneration and wound healing processes, both in vivo and in vitro. The piezoelectricity of the dermal patch resulted in enhanced expression of various markers and upregulated genes associated with important functions [90]. These included PCNA (proliferating cell nuclear antigen, involved in cell proliferation), SM α-actin (smooth muscle α-actin, associated with myofibroblast differentiation), TGF-β receptor (transforming growth factor beta receptor), Collagen III (providing initial wound structure and support for subsequent healing events), Collagen IV (promoting keratinocyte migration), as well as upregulated genes such as CD68 (related to macrophage function and inflammation), VEGF (vascular endothelial growth factor, involved in inflammation), integrin α5, TGF-β, and CD99 (associated with angiogenesis). Additionally, the phosphorylation of Akt, PI3K, ERK1/2, and Rho-GTPase was promoted [91].

Overall, the piezoelectric patches demonstrated significant improvements in migration, metabolic activity, differentiation, and electrotaxis of cultured fibroblasts, keratinocytes, and macrophages, ultimately leading to accelerated skin regeneration. Another study showed the positive effects of combining heating and piezoelectricity on wound healing, as depicted in Figure 6 [92].

## 8. Piezoelectric Devices for Biomedical Applications

### Wearable Devices

Wearable electronic devices have become vital for biomedical monitoring due to their advantages, including reduced weight, enhanced flexibility, and seamless integration with the human body. These devices serve various purposes, ranging from monitoring heart rate to measuring cortisol levels [93,94,95,96,97]. However, their effectiveness is often hindered by limitations in power supply technology [98]. To overcome this challenge, the integration of piezoelectric materials for energy harvesting in future wearable devices is of significant importance. Piezoelectric materials have the ability to convert mechanical energy into electrical energy, making them highly suitable for this purpose.

**Figure 7:**
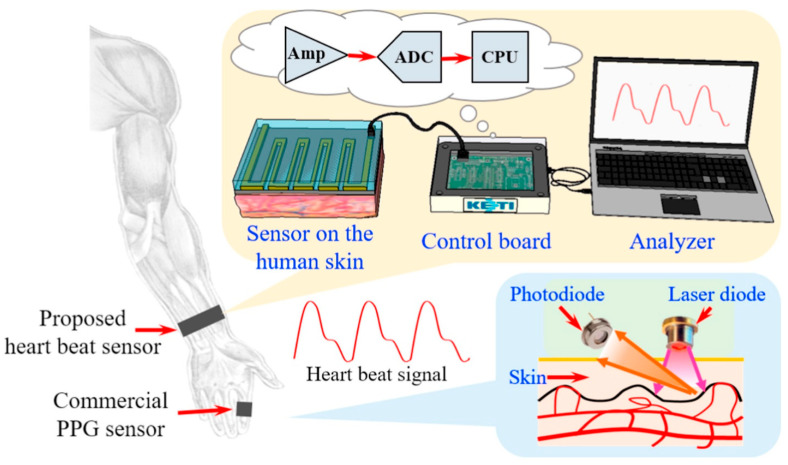
Polymer-based strain-gauge sensor for smart devices using heartbeat detection [95].

Conventional techniques, such as tape casting, used for fabricating piezoceramics, result in rigid materials and limited possibilities for creating intricate 2D structures. In the context of wearable applications, it is crucial for piezoelectric materials to exhibit flexibility, stretchability, and the ability to be fabricated with fine microscale resolution. Microstructured piezoelectric materials are particularly attractive as they can be interconnected to form arrays using soft copper wire. These microstructured arrays enable more efficient energy harvesting and can conform to the stretchable and flexible surface of the human body, thereby enhancing practicality [8]. As shown in Figure 7, the detection circuit, based on a Wheatstone bridge design with four resistors, utilized a strain gauge sensor patterned on both sides. Two additional resistors were incorporated into a custom control board, which also included components for voltage regulation, signal amplification, and data conversion. This system allowed for connection to a PC with verification software for sensing signals.

Polyvinylidene fluoride (PVDF), known for its inherent flexibility, low acoustic impedance, and excellent chemical stability, plays a significant role in the development of flexible energy harvesting structures for wearable devices [99]. Three-dimensional printing offers notable advantages in the production of piezoelectric materials, allowing for rapid manufacturing while preserving the desired piezoelectric properties of the material. This renders 3D printing a promising technique for fabricating piezoelectric materials intended for use in wearable devices.

Flexible piezoelectric polymers based on physical vapor deposition (PVD) were utilized to create rugby ball-shaped piezoelectric energy harvesters (PEH) [43]. These energy harvesters were fabricated using a 3D-printed flexible multilayer b-phase PVDF-TrFE copolymer with a high d33 value. The performance of vibration energy harvesting was compared among different device types, including one-layer flat PEH, six-layer flat PEH, one-layer flat PEH made of a-PVDF, one-layer rugby ball-shaped PEH, and six-layer rugby ball-shaped PEH. Results showed that the six-layer rugby ball-shaped PEH exhibited the highest output voltage (88.62 V) and power density (16.41 mW cm^−2^) at 10 Hz. The unique structure of the rugby ball shape contributed to this exceptional performance. Additionally, the harvested AC voltage was rectified to generate DC power, which could be used to charge a Li-ion battery and power LEDs [43]. Therefore, the application of 3D printing enables the efficient manufacturing of PEH devices with high power density, making it an attractive technique to meet the power requirements of self-powered wearable electronics in future applications.

Further advancements in 3D printing of piezoelectric wearables were demonstrated by Li et al. They fabricated a self-powered sensor using a 3D-printed hydrophobic surface-functionalized BTO/PVDF composite film [50]. The functionalization process improved the bond between BTO and PVDF, increasing the amount of β-phase PVDF in the composite. The resulting sensors generated a 30 V output response to a pressure input of 500 kPa. These 3D-printed BTO/PVDF composite sensors were integrated into protective sports gear for Korean Taekwondo martial arts to monitor human movement [50]. Through experiments, the voltage generated by the smart-sensor protective gear was utilized to measure the magnitude of striking force. The self-powered sensor array, implemented using the 3D-printed BTO/PVDF sensors, converted input pressure into electrical power, enabling the monitoring of human motion and mapping of motion patterns without the need for a standalone energy source. This technique holds promise for harvesting biomechanical energy in standalone biomedical monitoring systems.

## 9. Ultrasound Devices

Ultrasonic devices have found extensive applications in the field of biomedical devices, particularly in energy harvesting and bioimaging, due to their non-invasive nature [8,9,100,101]. The performance of ultrasonic devices is largely determined by the piezoelectric materials used in their internal structure, which affect sensitivity, electrical impedance, and bandwidth [8]. Optimizing these parameters is crucial for enhancing device functionality, and the emerging technology of 3D printing offers promising opportunities to selectively optimize these performance factors and fabricate complex shapes. Consequently, there has been growing research interest in 3D printing of piezoelectric materials [100].

One 3D printing method called Mask Image Projection-based Stereolithography (MIP-SL) has been employed to fabricate ultrasonic devices [101]. The 3D-printed concave piezoelectric element (PF-CPE) was assembled into an ultrasound transducer housing for ultrasonic imaging applications. The transducer exhibited an electromechanical coupling factor (kt) of 0.474 with a peak-to-peak pulse-echo response magnitude of 0.35 V. The transducer demonstrated a line spread function resolution of 240 µm in the axial direction and 770 µm in the lateral direction. The fabricated device was used to image a porcine eyeball, revealing the cornea and other layers with promising potential for ultrasonic imaging applications [9].

**Figure 8:**
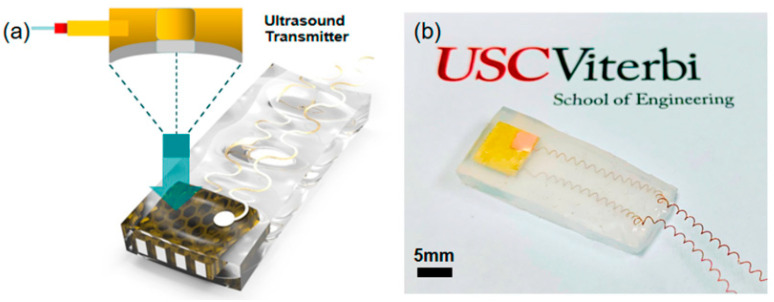
(**a**) Schematic and design of the ultrasonic device. (**b**) Optical image of the fabricated device [101].

In another study utilizing stereolithography (SLA), a 3D-printed BTO ceramic ultrasound transducer was developed with a focused ultrasonic array, operating at a center frequency of 1.4 MHz, 40% fractional bandwidth, and 50 dB insertion loss. The printed array exhibited a pulse-echo response magnitude of 0.28 V, demonstrating sufficient sensitivity for ultrasonic imaging [8]. Chen et al. showcased a 3D-printed annular piezoelectric array structure that modified the acoustic beam, leading to improved spatial resolution in ultrasonic imaging. The printed array achieved a lateral resolution (beam width) of less than 1.1 mm, with a tunable focal depth ranging from 5.6 mm to 8 mm. Furthermore, Zeng et al. employed MIP-SL to fabricate ultrasonic devices with complex honeycomb structures for ultrasonic sensing applications. By adding low-permittivity epoxy, the 3D-printed BTO honeycomb structure served as a piezoelectric composite, as shown in Figure 8. The fabricated device, encapsulated in silicone rubber, converted received ultrasound waves into electrical signals, generating a maximum value of 180 mVpp and an output power of 9 nW. These 3D-printed structures with intricate geometries show promise for wireless energy harvesting applications and warrant further investigation [9].

## 10. Polymer Piezoelectric Based Nanogenerator (PPENG)

Numerous piezoelectric nanogenerators (PENG) have found applications in various biomedical fields, such as the circulatory system, neural system, stem cell differentiation, and water decontamination, biodegradable electronics, and ongoing research includes drug delivery, voice recognition, and biomonitoring. The development and implementation of self-powered biomedical devices are poised to have a significant impact on the healthcare industry. Ravikumar et al. created a piezoelectric nanogenerator (PENG) using a metal-phenolic coordination framework (MPCF) and a polymer composite. The composite consisted of copper oxide nanosphere-tannic acid (CuO-TA) and copper-tannic acid (Cu-TA) nanocubes embedded in MPCF and PVDF. They constructed the device by sandwiching a 2 × 2 cm MPCF composite film between two copper electrodes, which were then encapsulated in PDMS. The researchers compared two different composites, CuO-TA/PVDF and Cu-TA/PVDF, and observed a significant difference in performance [102]. The CuO-TA/PVDF composite generated an output voltage of 40.20 V with a current of 4.40 µA, while the Cu-TA/PVDF composite produced an output voltage of 8.90 V with a current of 3.80 µA. The device was used for physiological monitoring and the harvesting of biological energy.

The authors specifically explored the potential of the CuO-TA/PVDF-based PENG as a physiological monitor for heart disease prevention. They claimed that real-time monitoring of heart rate and arterial pulse could help prevent heart attacks [103]. To evaluate the device’s effectiveness, they attached it to a healthy 22-year-old person and monitored their heart rate before and after exercise. The recorded values were approximately 76 and 145 beats per minute (BPM), respectively. Comparing the results with a commercially available device, they found the measurements to be quite accurate, demonstrating the sensitivity and applicability of their fabricated device. Additionally, the authors integrated the PENG with the Internet of Things (IoT) to enhance its application performance, enabling users to remotely access their pulse rate from anywhere. All fabricated devices were flexible, self-powered, and sufficiently sensitive to differentiate between normal and accelerated pulse rates, making PENGs suitable for various health monitoring applications. Another development in this field was a wireless, self-powered heart sensor created by Rana et al. They demonstrated the fabrication of a self-powered, auto-operated wireless sensor based on porous carbon. The device exhibited high reliability, making it suitable for diverse applications, even in challenging environments. It achieved an output current of 22 µA and a peak-to-peak voltage of 84.5 V while being environmentally friendly [104]. The researchers constructed an entire self-powered network for sensing purposes.

Piezoelectric nanogenerators (PENGs) have been explored for implantable applications due to the biodegradable properties of piezoelectric materials. Wu et al. developed a PENG to enhance nerve tissue repair [105]. They constructed a device using KNN, poly(L-lactic acid) (PLLA), and a proposed material called Poly(3-hydroxybutyrate-co-3-hydroxyvalerate) (PHBV). Poly(lactic acid) (PLA) or poly(ε-caprolactone) (PCL) was used for encapsulation. The fabricated device was biodegradable and capable of harvesting energy from ultrasounds. The researchers evaluated its functionality in different environments, such as water and cell culture, to confirm its biodegradability. Moreover, the device was successfully implanted in a laboratory rat for 3 weeks without causing any organ damage. They concluded that the fabricated devices were safe for both in vitro and in vivo applications. The device effectively delivered electrical stimuli, promoting nerve repair and monitoring without the need for rectifiers. Azimi et al. introduced a self-powered cardiac pacemaker based on the piezoelectric polymer PVDF. PVDF is a safe and biocompatible material [106]. Traditional pacemakers rely on batteries with a limited lifespan, requiring replacement. Self-powered devices offer a solution to this limitation. In their study, the authors fabricated a PVDF/ZnO/rGO-based PENG using the electrospinning method to power a pacemaker. The self-powered pacemaker was tested on an animal model. Following implantation on the lateral wall of the left ventricle of a dog, the PENG harvested 0.487µJ of energy with each heartbeat. This development represents a significant step toward the fabrication of smart implant devices and showcases the future potential of self-powered implants. Shrama et al. designed an electric stimulator for accelerating wound repair using PVDF. They created a piezoelectric hydrogel component composed of carbonized polydopamine/polydopamine/polyacrylamide, combined with an electrospun PVDF membrane [107]. The hydrogel exhibited excellent mechanical strength, supporting activities such as walking and stretching. The fabricated film stimulated cell growth through electric stimulation generated by body movements and provided protection against bacterial infections in underlying wounds.

### 11. 3D Printing of Polymer Piezoelectric Materials

The utilization of piezoelectric materials has resulted in significant advancements in the biomedical engineering field. When it comes to additive manufacturing (AM), the initial crucial step is to select appropriate piezoelectric materials that are compatible with 3D printing. This selection process is heavily influenced by the intended application of the device, as it directly impacts its mechanical and electrical capabilities [108,109]. Specifically, the 3D-printed piezoelectric material should possess piezoelectric coefficients and acoustic properties that are equal to or superior to those achieved with traditional fabrication methods.

**Figure 9:**
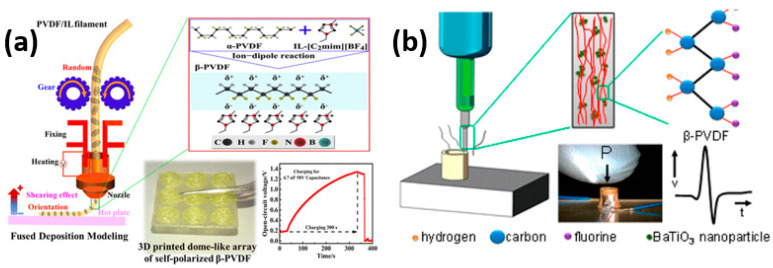
(**a**) 3D printing of PVDF nanocomposite-based structures using a solvent-evaporation-assisted method. (**b**) Image of the cylindrical sensor subjected to a finger-tap test. Piezoelectric voltage generated by the cylindrical sensor in response to five successive finger taps. Diagram illustrating the suggested process that enhances the β-phase fraction in PVDF through the incorporation of fillers. Reproduced with permission [115].

The advancement of flexible electronics necessitates the utilization of materials that retain their electrical and structural properties even when subjected to twisting and stretching [110,111,112]. Traditional piezoceramics have been limited in flexible device applications due to their brittleness and rigidity. Consequently, there has been growing interest in flexible piezoelectric polymers that exhibit excellent piezoelectric properties, particularly in the biomedical field. Among these polymers, PVDF (polyvinylidene fluoride) and its copolymers have emerged as the most prominent and widely used piezoelectric functional polymers [29,113]. PVDF is synthesized through the polymerization of vinylidene difluoride and exists in various crystal phases, including α, β, γ, and δ phases [112,114]. Notably, the β phase of PVDF demonstrates exceptional ferroelectric and pyroelectric properties [115]. As a result, piezoelectric polymers have found extensive application in 3D printing for the fabrication of thin layers and films used in device and sensor production. Liu et al. used ionic liquid-modified FDM 3D printing forces for direct PVDF printing to maintain the β-crystalline phase of PVDF. As depicted in Figure 9a, more than 98% of the β-phase is retained after printing with a 4.7 times higher piezoelectric output voltage as compared to the flat PVDF [115]. In another study, the addition of barium titanate nanoparticles was studied on the effects of nucleation of piezoelectric polymorph in 3D printable polyvinylidene fluoride (PVDF). With a piezoelectric coefficient, d31, of 18 pC N^−1^, the nanocomposite formulation developed after a thorough analysis of composition and manufacturing methods is comparable to the kind of poled and stretched commercial PVDF film sensors [109]. The effectiveness of the construction method is demonstrated by a 3D contact sensor that can produce up to 4 V in response to light finger taps. Piezoelectric nanocomposites can be 3D printed in one step to create ready-to-use, intricately formed, flexible, and lightweight piezoelectric devices, as shown in Figure 9b.

Similar to the 3D printing of piezoceramics, PVDF can also be employed in SLA (stereolithography). In a representative study [116], PVDF powder was mixed with a photopolymer resin in N, N Dimethylformamide (DMF) using a magnetic stirrer. The mixed slurry was then subjected to photocuring in an SLA 3D printer, producing samples with a layer thickness of 100 µm [99]. The optimization of the 3D printing process focused on the weight ratio of PVDF to DMF solvent, aiming to achieve a homogeneous dispersion of PVDF molecular chains within the photopolymer resin. Increasing the weight ratio of PVDF to DMF resulted in a coarser surface and reduced PVDF agglomeration in the 3D-printed PVDF. This outcome was likely due to the precipitation polymerization of PVDF within the photopolymer resin. Electrical poling was carried out at room temperature with an applied voltage of 3 MV m^−1^ for 15 h, resulting in a piezoelectric coefficient (d31) of 0.014 pC N^−1^ for a 2 wt% PVDF/photopolymer resin composition. Another study demonstrated that 3D-printed PVDF with multiple layers exhibited a higher piezoelectric coefficient of 130 pC N^−1^. Additionally, material extrusion AM (MEAM) was investigated for the fabrication of 3D-printed PVDF using homopolymer PVDF resin pellets extruded layer by layer at an optimized temperature of 210 °C. Tensile tests conducted on the 3D-printed samples showcased promising mechanical properties. To enhance the conventional poling process, which typically requires high voltage and a long duration, PVDF-co-hexafluoropropylene (PVDFHFP) was fabricated using a DLP printing process and an in situ poling system. This innovative printing technique reduced the total processing time by a factor of 10 and yielded a printed PVDF film with a piezoelectric coefficient of 42 pC N^−1^ [117]. However, further improvements are needed to match the piezoelectric properties of 3D-printed PVDF with those of traditional piezoceramics. Although several 3D printing methods for PVDF production exist, the selection of piezoelectric polymers suitable for 3D printing in biomedical applications remains limited. Consequently, piezoelectric polymers such as PLA (polylactic acid) or PVF (polyvinyl fluoride), will be further studied in 3D printing process for biomedical applications.

## 12. Advantages and Challenges Associated with Utilizing Polymer Piezoelectric Materials in Biomedical Applications

Polymer piezoelectric materials offer several advantages and face certain challenges when utilized in biomedical applications.

Advantages: Flexibility and Conformability: Polymer piezoelectric materials are highly flexible and can conform to irregular shapes and surfaces. This property makes them suitable for applications where conformability to the human body is required, such as wearable devices or implantable sensors.

Biocompatibility: Many polymer piezoelectric materials exhibit good biocompatibility, meaning they are compatible with living tissues and do not cause adverse reactions or toxicity. This property is crucial for biomedical applications to ensure the safety and effectiveness of the devices.

Low Cost and Easy Fabrication: Polymer piezoelectric materials can be manufactured at a relatively low cost compared to traditional ceramic piezoelectric materials. They can be synthesized using simple techniques like solution casting or electrospinning, enabling large-scale production and potential commercialization.

Lightweight: Polymer materials are generally lightweight, which is advantageous for wearable or implantable devices. Their low weight contributes to patient comfort and reduces the burden on the body.

Tunable Properties: Polymer piezoelectric materials can have their properties tailored to specific application requirements by adjusting the chemical composition or processing parameters. This tunability allows customization for different biomedical applications, optimizing their performance and functionality.

Challenges: Lower Piezoelectric Performance: Polymer piezoelectric materials typically have lower piezoelectric coefficients compared to their ceramic counterparts. This limitation affects the sensitivity and efficiency of energy conversion in devices utilizing these materials, requiring careful design considerations to overcome this challenge.

Mechanical Durability: Some polymer materials may exhibit reduced mechanical durability and fatigue resistance, limiting their long-term reliability in biomedical applications. Frequent mechanical stress or cyclic loading can lead to material degradation, affecting the performance and lifespan of the device.

Stability and Aging: Polymer materials are susceptible to environmental factors such as temperature, humidity, and chemical exposure. They may undergo degradation over time, leading to changes in their piezoelectric properties and overall performance. Ensuring long-term stability and reliability of polymer-based devices requires appropriate material selection and protective measures.

Processing Constraints: The processing techniques for polymer piezoelectric materials often require careful control of parameters such as temperature, solvent choice, and curing conditions. These constraints may limit the fabrication options and add complexity to the manufacturing process, impacting scalability and cost-effectiveness.

Integration Challenges: Integrating polymer piezoelectric materials into biomedical devices and systems can present challenges due to their electrical and mechanical interfaces. Ensuring the reliable electrical connections, robust packaging, and compatibility with other components require specialized design and engineering solutions.

In summary, while polymer piezoelectric materials offer advantages such as flexibility, biocompatibility, and low cost, they also face challenges related to lower piezoelectric performance, mechanical durability, stability, processing constraints, and integration. Addressing these challenges through material engineering, device design optimization, and manufacturing advancements will further enhance the feasibility and effectiveness of polymer piezoelectric materials in biomedical applications.

## Data Availability

Not applicable.
